# Decreased Astrocytic CCL2 Accounts for BAF-312 Effect on PBMCs Transendothelial Migration Through a Blood Brain Barrier in Vitro Model

**DOI:** 10.1007/s11481-021-10016-5

**Published:** 2021-10-02

**Authors:** Simona F. Spampinato, Sara Merlo, Giuseppe Costantino, Yasuteru Sano, Takashi Kanda, Maria Angela Sortino

**Affiliations:** 1 Department of Biomedical and Biotechnological Sciences, University of Catania, Catania, Italy; 2 Ph.D. Program in Neuroscience and Education, DISCUM, University of Foggia, 71121 Foggia, Italy; 3 Department of Neurology and Clinical Neuroscience, Yamaguchi University Graduate School of Medicine, Ube, Yamaguchi, Japan

**Keywords:** Multiple sclerosis, Siponimod, Transmigration, Astrocytes, Sphingosine-1-phosphate

## Abstract

Disruption of the blood brain barrier (BBB) is a common event in several neurological diseases and in particular, in multiple sclerosis (MS), it contributes to the infiltration of the central nervous system by peripheral inflammatory cells. Sphingosine-1-phosphate (S1P) is a bioactive molecule with pleiotropic effects. Agonists of S1P receptors such as fingolimod and siponimod (BAF-312) are in clinical practice for MS and have been shown to preserve BBB function in inflammatory conditions. Using an in vitro BBB model of endothelial-astrocytes co-culture exposed to an inflammatory insult (tumor necrosis factor-α and interferon-γ; T&I), we show that BAF-312 reduced the migration of peripheral blood mononuclear cells (PBMCs) through the endothelial layer, only in the presence of astrocytes. This effect was accompanied by decreased expression of the adhesion molecule ICAM-1. BAF-312 also reduced the activation of astrocytes, by controlling NF-kB and NLRP3 induction and preventing the increase of proinflammatory cytokine and chemokines. Reduction of CCL2 by BAF-312 may be responsible for the observed effects and, accordingly, addition of exogenous CCL2 was able to counteract BAF-312 effects and rescued T&I responses on PBMC migration, ICAM-1 expression and astrocyte activation. The present results further point out BAF-312 effects on BBB properties, suggesting also the key role of astrocytes in mediating drug effects on endothelial function.

**Graphical abstract:**

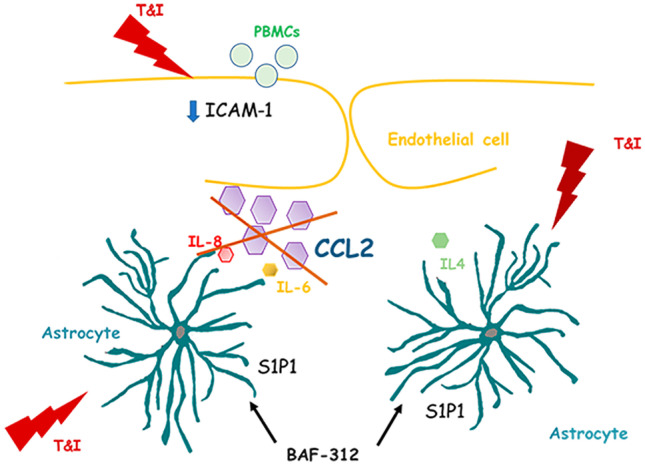

## Introduction

The Central Nervous System (CNS) has long been considered an immunity sanctuary, where, in physiological conditions, both pathogens and cells of the immune system are not allowed. In truth, this vision has been long overcome (Carson et al. 2006). Microglial cells do in fact provide a persistent scanning and cells of the immune system can reach the CNS. The access of immune cells is however carefully limited to the ones necessary to patrolling, and when their access in the CNS is uncontrolled, important dysfunctions may occur (Engelhardt and Ransohoff 2005). The blood brain barrier (BBB) is an import structure necessary to prevent this event. The lack of fenestrations, the strict connections between cells and the very limited expression of adhesion molecules are the main and peculiar features of the endothelial cells at the BBB. These characteristics are further strengthened by the presence of astrocytes, whose role in maintaining and reinforcing BBB properties has been fully proved (Abbott et al. 2010; Obermeier et al. 2016). CNS affections may involve and often be the consequences of BBB damage, as observed in diseases such as Alzheimer’s disease, ischemia and multiple sclerosis (MS) (Obermeier et al. 2016). In MS BBB stability may be transiently but recurrently affected: increased barrier permeability has been linked to disease activity so that the leakage of the tracer gadolinium is used to detect active inflammatory lesions (Molyneux et al. 1998; Saade et al. 2018). Moreover, both CD4+ and CD8+ T lymphocytes easily transmigrate through the BBB due to the modification of adherens junctions (AJ) and tight junctions (TJ), as well as to increased expression of adhesion molecules that facilitate endothelial-immune cells interaction and the migration through cell-to-cell junctions (intercellular migration) or through endothelial cells (transcellular migration). Of note, extravasation of auto-aggressive T-cells through the barrier induces demyelination and axonal loss, leading to progressive disability (Ortiz et al. 2014). The activation of proinflammatory pathways, such as NF-κB, and the release of proinflammatory factors, such as interleukin (IL) -6 and chemokines, like CCL2, also facilitate BBB breakdown (Sheikh et al. 2020; Xiao et al. 2020). In physiological host defense conditions, CCL2 binds to its specific receptor, CCR2, mainly found on monocytes, leukocytes and T cells (Carrillo-de Sauvage et al. 2012; Gschwandtner et al. 2019), promoting extravasation of effector cells. However, in pathological conditions, like in MS, its aberrant expression exacerbates inflammation (Gschwandtner et al. 2019). Acting on its receptor CCR2, the chemokine induces TJ redistribution, AJ disruption (Roberts et al. 2012; Roblek et al. 2019) and actin cytoskeleton reorganization (Stamatovic et al. 2003, 2005; Xu et al. 2021), facilitating leukocytes infiltration and increasing barrier permeability.

Several molecules have been approved to treat MS and they operate using different mechanisms. Recently, the role played by sphingosine-1-phosphate (S1P) modulators has emerged. S1P is a bioactive mediator that interacts with a class of G protein-coupled receptors (S1P1-S1P5). S1P receptors are widely expressed in our body, and in particular S1P1 is responsible for the egress of naive and central memory T cells from the lymph nodes (Sica et al. 2019). The superagonist FTY-720 (FTY; fingolimod), by downregulating S1P1 expression on lymphocyte surface, has been proved capable of dropping the number of circulating autoreactive lymphocytes, thus reducing MS relapses. Very recently, another S1P1 modulator, BAF-312 (BAF; siponimod), has been approved for the treatment of secondary progressive form of the disease. As mentioned, the expression of S1P receptors is not only limited to the cells of the immune system. Both endothelial cells and astrocytes, main cellular components of the BBB, do express them. In particular it has been proved that both FTY and BAF preserve barrier properties in an in vitro model of BBB. FTY, acting through astrocytes, reduced endothelial damage (Spampinato et al. 2015) while BAF, acting on both S1P1 and S1P5, reduced endothelial permeability induced by an inflammatory stimulus (Spampinato et al. 2021). Here, we further investigated whether BAF could also affect transendothelial migration of peripheral blood mononuclear cells (PBMCs), using an in vitro model of BBB, recapitulating an inflammatory condition.

## Materials and Methods

*Reagents*. Collagen-I-rat tail was provided by Corning (Milan, Italy). Tumor necrosis factor-α (TNFα), interferon-γ (IFNγ), CCL2 and CXCL12 were purchased from Peprotech (London, UK), RS-504393 was from Tocris Bioscience (Bristol, UK), and JTE-013 from Sigma-Aldrich (St Louis, MO, USA). BAF-312 (BAF) and NIBR-0213 (NIBR) were provided by Novartis Pharma (Basel, Switzerland).

*Cell cultures*. Cell lines used to simulate the BBB are adult human immortalized cells. Both the endothelial cell line, TY-10 cells, brain microvascular endothelial cells, and hAST, astrocytic cells, are transfected with a plasmid expressing temperature sensitive Simian virus-40 large T-antigen and the catalytic subunit of human telomerase, as previously described (Haruki et al. 2013). Both cell lines were developed at Yamaguchi University (Japan), in the labs of Dr. Sano and Kanda. hAST were grown in astrocyte medium containing 2% heat-inactivated Fetal Bovine Serum (FBS), astrocyte growth supplement and penicillin/ streptomycin solution, as provided with the Astrocyte media kit (Clinisciences, Nanterre, France). TY-10 cells were grown in MCDB-131 media (Thermo Fisher Scientific, Milan, Italy), supplemented with EGM-2 SingleQuots (Lonza, Basel, Switzerland) and 20% heat-inactivated FBS (Thermo Fisher Scientific). All cell culture plastics were from BD Falcon (Milan, Italy). For all experiments, cell lines at passage between 18 and 24 were used. Both cell lines grew at 33 °C for two days and then were transferred to 37 °C, where they exhibited growth arrest and differentiation. After two days at 37 °C, cells were exposed to treatments. Treatments were done in astrocyte medium, and all experiments were performed in the presence of S1P2 antagonist JTE-013 (1 µM). Treatment with cytokines (TNFα, 5 IU, and IFNγ, 10 IU; T&I) was maintained either for 30 min or 24 h, according to the experimental protocol. Endothelial monocultures or endothelial/astrocytes co-cultures, when required by experimental design, were pretreated with NIBR (1 µM) for 30 min or BAF (100 nM) for 15 min prior to T&I exposure and were then maintained in association with the cytokines for further 30 min or 24 h, according to experimental protocol. CCL2 (100 ng/ml) was applied 15 min prior to BAF and, when required, cultures were pre-exposed to the highly selective CCR2 antagonist RS-504393 (1 µM), 15 min prior to chemokine exposure. To obtain astrocyte conditioned medium (ACM), astrocytes were exposed to either vehicle (ACM ctr), to T&I (5 IU and 10 IU; ACM T&I) or to T&I + BAF (100 nM, ACM B + T&I) for 6 h. Cultures were then washed and incubated with fresh medium for further 18 h. ACM was then collected and transferred on endothelial cultures that were exposed to T&I (5 IU and 10 IU respectively) for 24 h. This particular paradigm was chosen in order to allow exposure of astrocytes to T&I and BAF for a reasonable time to induce astrocytic effects before removal, and has been already described (Spampinato et al. 2017). Cryopreserved human Peripheral Blood Mononuclear Cells (hPBMC) were provided by Lonza. They were stored in nitrogen until thawing, according to manufacturer protocols. Thirty million thawed PBMCs were grown at 37 °C for two days in a t75 cm^2^ flask, in phenol red-free RPMI medium supplemented with 10% FBS, glutamine and non-essential amino acids (all from Thermo Fisher Scientific).

*Migration Assay*. The protocol for static transmigration assay has been previously described (Spampinato et al. 2020). For the assay we used 6.5 mm polycarbonate membrane cell culture inserts with 8.0 µm pore (Corning Transwell). Inserts were previously coated using collagen-I-rat tail on both the abluminal and luminal side. hAST (3 × 10^5^ cells per wells) were seeded on the abluminal side of the membrane. After attachment, inserts were flipped and TY-10 (5 × 10^5^ per membrane), seeded on the luminal side. The ratio astrocytes/endothelial cells was of 1:1,6. Co-cultures were grown in astrocyte medium for 2 days at 33 °C and then kept for 2 days at 37 °C. Treatments were performed in astrocyte medium for 24 h at 37 °C. Before the assay, the apical endothelial layer was exposed to CXCL12 (50 ng/ml in medium) and incubated for 15 min at 37 °C. FBS 1% was used as chemoattractant in the abluminal side. hPBMCs (2.8 × 10^6^ cells per assay) were added on the top of the endothelial layer. The assay was performed at 37 °C in transendothelial migration buffer (TEM, RPMI 1640 without phenol red, 1% bovine serum albumin, Hepes, L-glutamine, Na-pyruvate, MEM non-essential amino acids, all from Thermo Fisher Scientific). After 18 h, migrated hPBMCs were recovered from the bottom chamber and counted.

*Western Blot*. Endothelial cell monocultures (5 × 10^5^ cells per wells) were plated on 6 wells MW plates. When co-cultured with endothelial cells, hAST (3 × 10^5^ cells per wells) were plated on 0.4 μm pores poly-carbonate membrane transwell inserts (Falcon). This setting allowed the passage of soluble factors between endothelial cells and astrocytes layer, but not their direct physical contact, thus also facilitating their isolation for western blot investigations. After treatments, all carried out in astrocyte medium, pellets were collected and lysed in RIPA lysis buffer (Sigma-Aldrich) supplemented with protease and phosphatase inhibitors. Forty μg of each sample were separated by sodium dodecyl sulfate PAGE and transferred to nitrocellulose membranes. Membranes were blocked with Blocker™ FL Fluorescent Blocking Buffer (Thermo Fisher Scientific). The following primary antibodies were used: anti-mouse ICAM-1 (1:800; Santa Cruz Biotechnology), mouse anti–pNF-kB (1:500, Cell Signaling), rabbit anti-NF-kB (1:500, Cell Signaling), rabbit anti-NLRP3 (1:300, ThermoFisher Scientific), mouse anti-GAPDH (1:800; Millipore), rabbit anti-β-actin (1:3000, Sigma-Aldrich). After incubation, membranes were processed for immunodetection using specific fluorescent AlexaFluor 488 Plus-conjugated secondary antibody (ThermoFisher Scientific) and IRdye 800 secondary antibody (Licor). Fluorescent signals were detected using IBright 1500 (Thermo Fisher Scientific). Band intensity was analyzed using the image processing software “ImageJ” developed by NIH and in the public domain.

*Quantitative Real-Time Polymerase Chain Reaction (PCR)*. Astrocytes (3 × 10^5^ cells per wells) were plated on 6 wells MW plates, while co-cultured endothelial cells (5 × 10^5^ cells per wells) were plated on 0.4 μm pores poly-carbonate membrane transwell inserts (Falcon), allowing the passage of soluble factors and facilitating astrocytes/endothelial selective isolation. Total RNA was extracted from astrocyte cell cultures using the RNeasy Plus Micro Kit (Qiagen). One μg of RNA was used for cDNA synthesis, using the Superscript-VILO kit (Thermo Fisher Scientific). Quantitative RT-PCR was performed with Rotor-Gene Q using QuantiNova SYBR Green PCR Kit (Qiagen). The melting curves obtained after each PCR amplification reaction confirmed the specificity of the 2-[N-(3-dimethylaminopropyl)-N-propylamino]-4-[2,3-dihydro-3-methyl-(benzo-1,3-thiazol-2-yl)-methyli-dene]-1-phenyl-quinolinium (SYBR Green assays). The following pairs of primers from Invitrogen (Thermo Fisher Scientific) were used: human CCL2F (CCC CAG TCA CCT GCT GTT AT), human CCL2R (AGA TCT CCT TGG CCA CAA TG), human IL8F (CTT GGC AGC CTT CCT GAT TT), human IL8R (TTC TTT AGC ACT CCT TGG CAA AA). The following primeQuantitec primers (Qiagen) were used: human IL-6 (QT00083720), human IL–4 (QT00012565) and human RPLP0 (QT00075012) that was used as an endogenous control. Expression fold changes were calculated by applying the 2 – ΔCt method.

*Immunocytochemistry*. TY–10 cells were plated on collagen-I-rat tail-coated coverslips. Astrocytes were plated on the transwell tissue culture inserts, and transferred on top of the endothelial monolayer. Co-cultures were checked for confluency and after two days were treated as indicated. After 18 h treatment, TY–10 cells were pre-fixed in 2% PFA in medium for 5 min at 4 °C and then fixed in 2% PFA in PBS at RT for 5 min. Cells were then exposed to 0.2% Triton X–100 in PBS for 5 min at 4 °C to allow membrane permeabilization. Primary antibody mouse anti-VE-Cadherin (1:100, Santa Cruz Biotechnology) was incubated overnight in the presence of 0.1% Triton X–100 at 4 °C. Secondary antibody, goat anti-rabbit AlexaFluor 488 (1:300, Thermo Fisher Scientific) was incubated for 45 min at 25 °C in in the presence of 0.1% Triton X–100. 4,6-Diamidino-2phenylindole (DAPI) was used for counterstaining. Slides were imaged using an epifluorescent Zeiss Observer.Z1 microscope equipped with the Apotome.2 acquisition system connected to a digital camera (Zeiss, Oberkochen, Germany).

*Statistical Analysis*. All data are expressed as mean ± SEM of 3–5 different experiments each run in duplicates or in triplicates as specified in the figure legends. Data were analyzed by one-way ANOVA, followed by Newman-Keuls test for significance. *P* < 0.05 was taken as the criterion for statistical significance.

## Results

### BAF-132 Modifies PBMCs Migration in a BBB Cellular Model

**Fig. 1:**
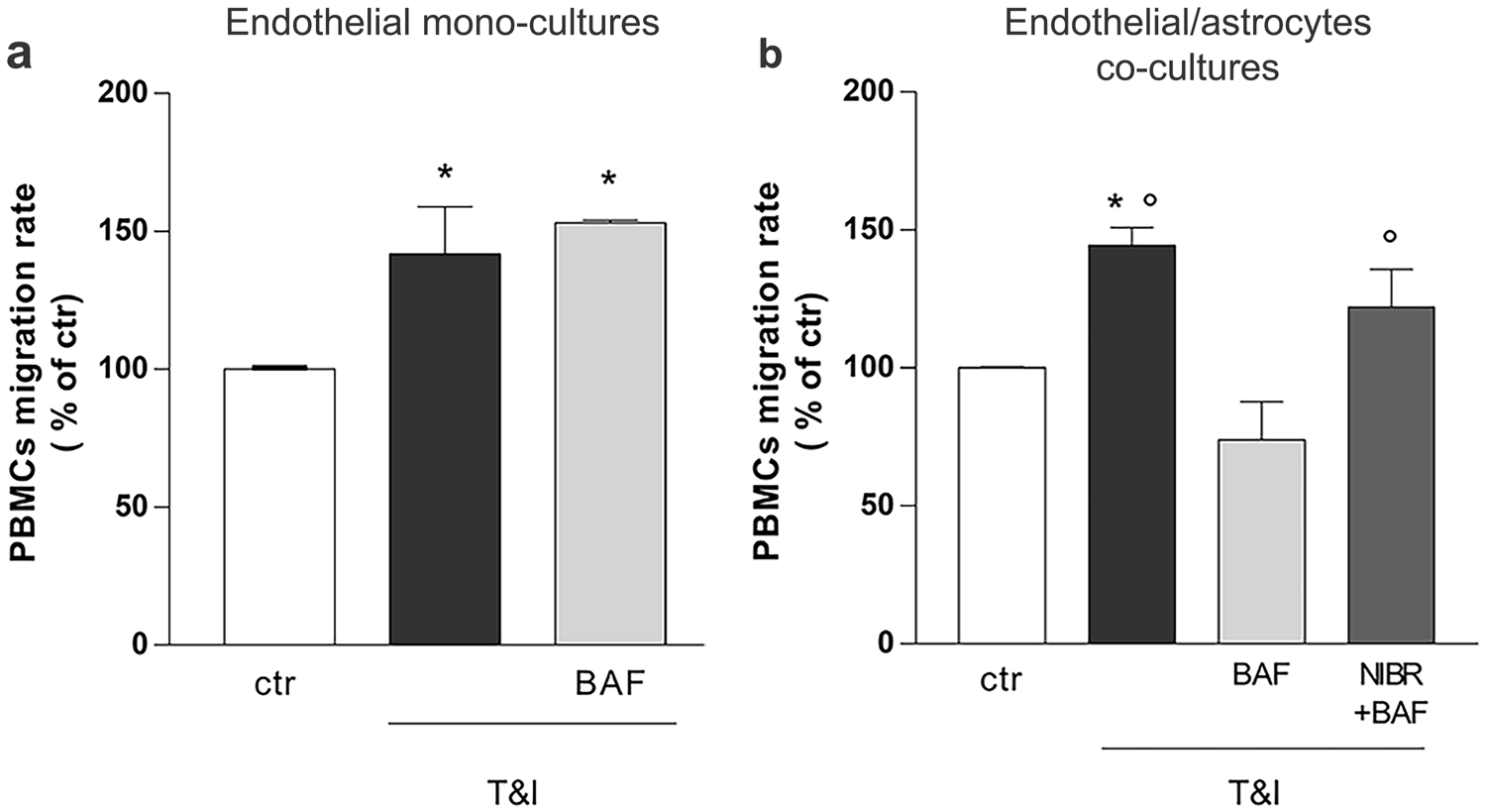
BAF-312 affects PBMCs migration through the in vitro BBB, only in the presence of astrocytes. To evaluate whether BAF exposure could modify T&I-induced PBMCs transmigration through the endothelial barrier, endothelial monocultures (**a**) and endothelial/astrocytes co-cultures (**b**) were exposed to inflammatory cytokines, TNFα (5 IU) and IFNγ (10 IU, T&I), for 24 h in the presence of BAF (100 nM) and NIBR (1 μM, b), that were added to the cultures respectively 30 min and 15 min prior to cytokines exposure. hPBMCs were then added on top of the endothelial layer and their ability to cross the in vitro barrier was evaluated after 24 h. PBMCs migration represents the ratio of migrated cells through the barrier, given a constant input, and are expressed as percentage of migration in control conditions. Data are mean ± SEM of 3 (**a**) or 4 (**b**) independent experiments, each run in duplicate. ∗ *p* < 0.05 versus control, °*p* < 0.05 versus BAF + T&I. Significance was assessed by one-way ANOVA followed by Newman–Keuls test

In order to evaluate BAF modulation of BBB properties, the ability of PBMCs to cross the in vitro barrier was investigated. hPBMCs (2.8 × 10^6^ cells/well) were added on top of a monolayer of TY-10 endothelial cells exposed for 24 h to a mix of inflammatory cytokines, TNFα and IFNγ (T&I; 5 IU and 10 IU, respectively). Cytokines significantly increased hPBMC migration rate, a response that was not modified when monocultures were pretreated with the S1P agonist BAF-312 (BAF; 100 nM, Fig. 1a). As better described in Material and Methods section, BAF was always added 15 min before T&I. This BAF concentration was chosen on the basis of preliminary experiments carried out using a range of BAF concentrations (10–300 nM) in this experimental paradigm (not shown) and on our own previous results (Spampinato et al. 2021). Conversely, when endothelial cells were co-cultured with astrocytes and exposed to the same treatments, pre-exposure to BAF completely prevented cytokine-induced hPBMC migration (Fig. 1b). S1P1 receptor activation was likely involved, as shown by blockade of BAF effect by the S1PR1 selective antagonist NIBR-0213 (NIBR, 1 µM, Fig. 1b).

**Fig. 2:**
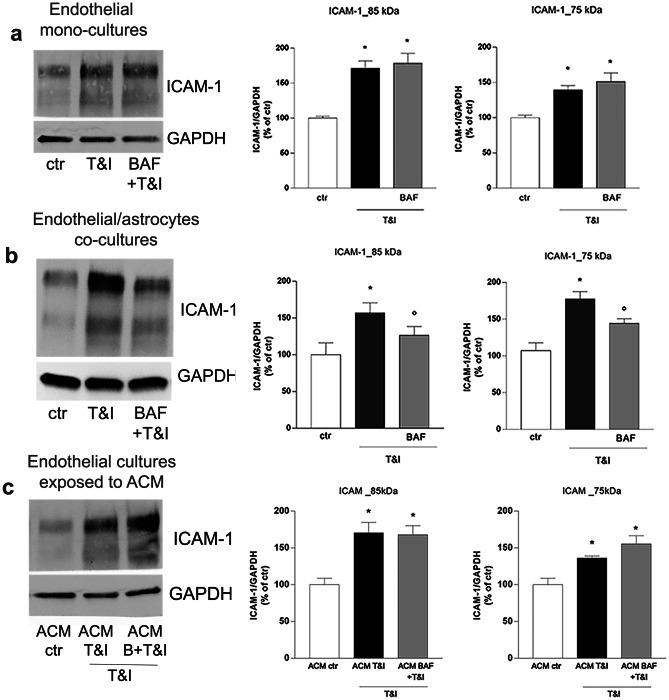
ICAM-1 expression is modulated by BAF-312 only in endothelial-astrocytes co-cultures. Endothelial cells, either cultured alone (**a**) or co-cultured with astrocytes (**b**), were pretreated for 15 min with BAF (100 nM), that was maintained for further 24 h in the presence of inflammatory cytokines, TNFα (5 IU) and IFNγ (10 IU, T&I). In (**c**) astrocytes were pre-exposed to BAF for 15 min prior to treatment with T&I for 6 h. Astrocytic cultures were then washed and medium replaced with fresh medium. After 18 h, astrocytic conditioned medium (ACM) was collected and transferred on endothelial cells, that were then exposed to T&I for further 24 h. The expression of ICAM-1 was evaluated by western blot analysis and representative blots and densitometric analysis of the two isoforms (85 and 75 kDa) are reported. Data are mean ± SEM of 5 (**a**, **b**) or 3 (**c**) independent experiments. ∗ *p* < 0.05 versus control, °*p* < 0.05 versus T&I. Significance was assessed by one-way ANOVA followed by Newman–Keuls test

In order to correlate the reduction of hPBMC migration induced by BAF to changes in endothelial properties, expression of the endothelial adhesion molecule ICAM-1 was investigated in the endothelial monocultures and in endothelial/astrocytes co-cultures. After 24 h exposure to T&I, expression of the adhesion molecule was induced in both conditions (Fig. 2a, b). In particular, we were able to identify two different ICAM-1 protein glycoforms (85 and 75 kDa) and they were both induced following cytokines exposure. BAF pretreatment did not modify ICAM-1 expression in endothelial mono-cultures (Fig. 2a), while it reduced T&I-induced over-expression of both the upper and lower molecular weight ICAM-1 isoforms in endothelial/astrocyte co-cultures (Fig. 2b). Based on these results, suggesting that astrocytes play a main role in the modulatory effect of BAF on barrier responses to T&I, the same experiments were carried out in endothelial monocultures grown in the presence of an astrocyte conditioned medium (ACM) (see Materials and Methods section for details). However, ACM derived from BAF-treated astrocytes, failed to affect T&I-induced ICAM-1 expression in endothelial cells (Fig. 2c).

### BAF-312 Modulates T&I-Induced Astrocyte Activation

**Fig. 3:**
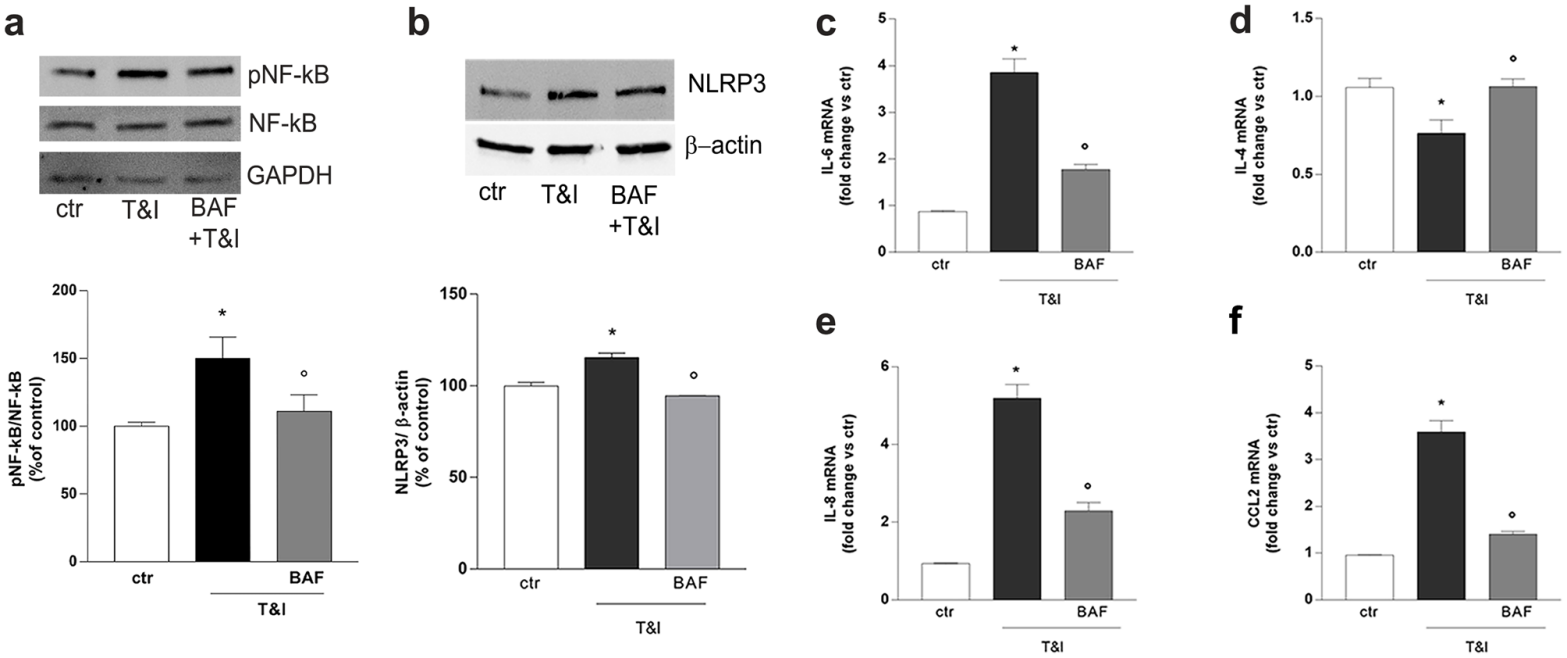
BAF-312 exposure decreases T&I-induced inflammatory state in astrocytes. To evaluate whether BAF modified T&I-induced astrocytic inflammatory states, endothelial/astrocytes co-cultures, pre exposed to BAF (100 nM) for 15 min, were further exposed to inflammatory cytokines TNFα (5 IU) and IFNγ (10 IU, T&I) for either 30 min (**a**) or 24 h (**b**–**f**). Astrocytic expression of phosphorylated NF-kB (**a**) and NLRP3 (**b**) was evaluated by western blot. mRNA levels of the inflammatory cytokine, IL-6 (**c**), anti-inflammatory cytokine, IL-4, (**d**) and of the chemoattractant chemokines, IL-8 (**e**) and CCL2 (**f**) were evaluated in astrocytes. Representative blots and densitometric analysis are reported (a, b). mRNA levels are reported as fold increase versus control (**c**–**f**). Data are mean ± SEM of 3 (**a**, **b**) or 5 (**c–f**) independent experiments. ∗ *p* < 0.05 versus control, °*p* < 0.05 versus T&I. Significance was assessed by one-way ANOVA followed by Newman–Keuls test

We then pointed our attention to the selective effects of BAF on astrocytes when they were co-cultured with endothelial cells. We first analyzed the ability of BAF to modify the state of activation of astrocytes induced by T&I. Indeed, astrocytic exposure to T&I (5 IU and 10 IU) for 30 min rapidly induced increased phosphorylation of NF-κB as shown by western blot analysis (Fig. 3a). In addition, treatment with the inflammatory cytokines for 24 h induced the expression of NLRP3 (Fig. 3b), one of the main constituents of the inflammasome, and accordingly, qRT-PCR revealed that mRNA levels of the inflammatory cytokine IL-6 were markedly induced while the anti-inflammatory cytokine IL-4 was down regulated (Fig. 3c, d). These effects on astrocytes were counteracted when co-cultures were pretreated (30 min before T&I exposure) with BAF (100 nM; Fig. 3a–d). Astrocytes release also chemoattractant chemokines, key mediators of peripheral cells migration, and in particular, in our model, mRNA transcription of both IL-8 and CCL2 were significantly induced after 24 h T&I treatment, effect that was again partially prevented when co-cultures were pre-exposed to BAF (Fig. 3e, f).

### Reduction of CCL2 is Responsible for BAF-312 Effects on Migration of Peripheral Cells Through the BBB

**Fig. 4:**
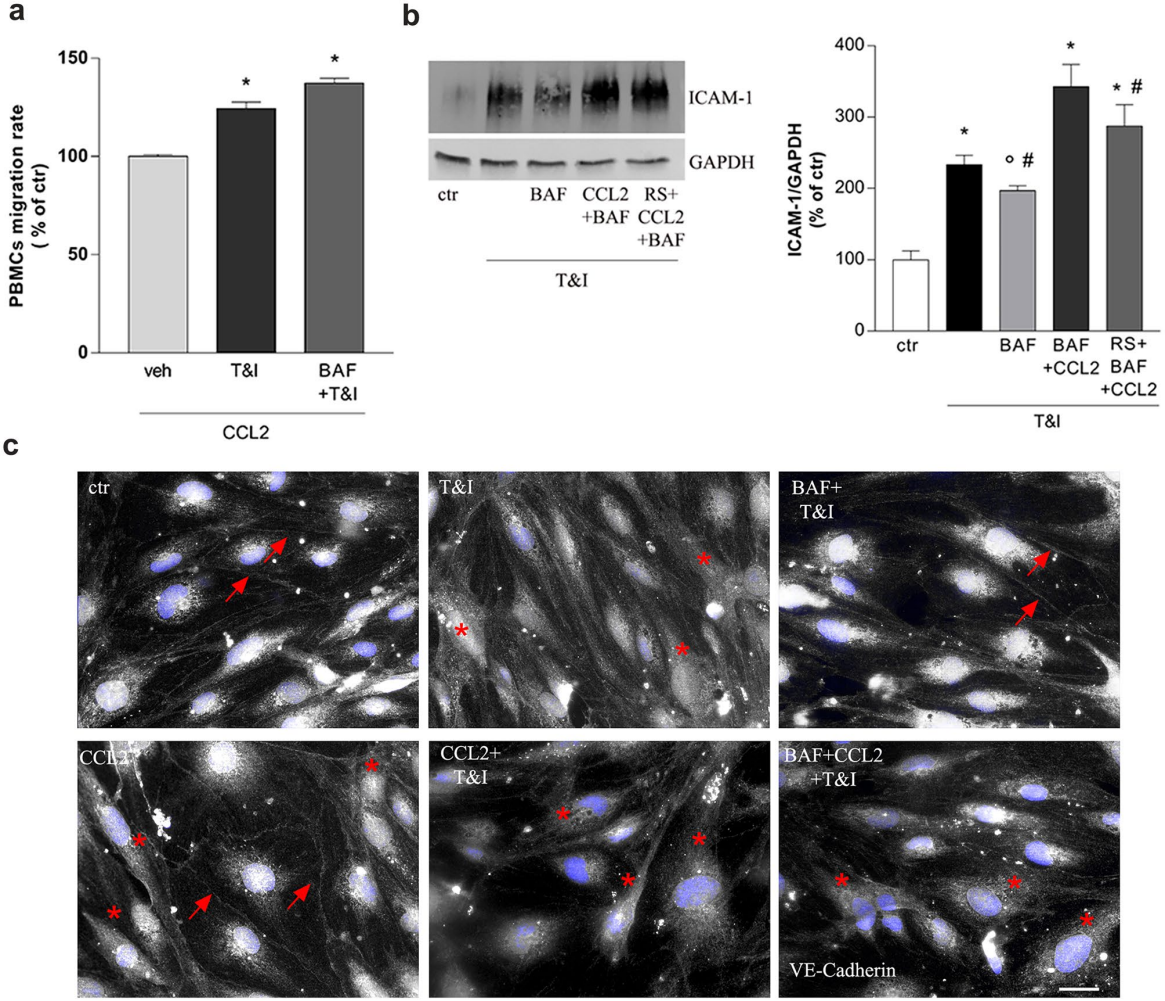
Exogenous CCL2 prevents BAF-312 effects on BBB properties. Endothelial/astrocytes co-cultures were pre-exposed to CCL2 (100 ng/ml) and BAF (100 nM) respectively 30 and 15 min prior to exposure to inflammatory cytokines TNFα (5 IU) and IFNγ (10 IU, T&I) for 24 h. hPBMCs were then added on top of the endothelial layer and their ability to cross the in vitro barrier was evaluated after 24 h (**a**). The CCR2 antagonist RS-504393 (1 μM) was applied 15 min prior to CCL2 on endothelial/astrocytes co-cultures that were exposed to inflammatory cytokines T&I for additional 24 h. Endothelial expression of ICAM-1 was evaluated by western blot analysis (**b**). In (**c**), cellular localization of the adherens junction VE-Cadherin was examined by immunocytochemistry analysis. hPBMCs migration represents the ratio of migrated cells through the barrier, given a constant input, expressed as percentage of migration in control conditions (**a**). Representative blots and densitometric analysis are reported (**b**). VE-cadherin is represented in gray, while 6–diamidino-2-phenylindole (DAPI) is used to counterstain nuclei. Asterisks (*) indicate proteins internalized in cytosolic compartments, in contrast to localization at cellular boundaries (indicated by arrows). Scale bar = 10 µm (**c**). Data are mean ± SEM of 3 (**a**, **b**) independent experiments. ∗ *p* < 0.05 versus control, °*p* < 0.05 versus T&I, # *p* < 0.05 versus CCL2 + BAF + T&I. Significance was assessed by one-way ANOVA followed by Newman–Keuls test

Since CCL2 has long been known to regulate endothelial cell permeability and to give an important contribution to BBB disruption, we wanted to verify whether changes of CCL2 could account for the observed BAF effects. Endothelial/astrocytes co-cultures pretreated with BAF (100 nM, 15 min) were exposed to T&I in the presence of CCL2 (100 ng/ml), added 10 min before BAF, and hPBMC migration through the barrier model was tested. CCL2 exposure, per se, did not modify hPBMCs migration rate (not shown), but it prevented BAF ability to counteract T&I-induced hPBMCs migration (Fig. 4a). Accordingly, CCL2, per se, did not affect the expression of ICAM-1 under basal conditions (not shown), but following T&I stimulation, in the presence of CCL2, BAF failed to reduce T&I-induced ICAM up-regulation (Fig. 4b). In addition, treatment with the CCR2 antagonist RS-504393 (1 μM) reduced CCL2 rescue of T&I-induced ICAM-1 expression (Fig. 4b). Finally, it is known that expression and localization of VE-cadherin is strongly involved in the leukocyte’s migration process. Accordingly, BAF (100 nM for 24 h) was able to prevent the redistribution of the AJ protein VE-Cadherin. In endothelial/astrocytes co-cultures, T&I caused translocation of VE-Cadherin from the cell membrane (Fig. 4c, arrows) to the intracellular compartment (Fig. 4c, asterisks). In contrast, in the presence of CCL2, VE-cadherin only partially moved towards the intracellular compartment (Fig. 4c, lower panel asterisks) and BAF failed to rescue its localization at the intercellular junctions (Fig. 4c, lower panels).

## Discussion

Data here reported highlight the substantial difference of BAF effect in mediating transendothelial PBMCs migration in endothelial monocultures and in endothelial/astrocytes co-cultures. Astrocytes mediate important endothelial properties at the BBB. They support endothelial cells physically and through the release of several factors capable of reinforcing barrier properties (Spampinato et al. 2019a). Astrocytes also affect endothelial response to noxious stimuli. In particular, we have previously observed that astrocytic mediators influence endothelial expression of the adhesion molecule ICAM-1, consequently affecting PBMCs migration through the endothelial barrier (Spampinato et al. 2019b). Accordingly, BAF effects on transendothelial migration here described were only observed when endothelial cells were co-cultured with astrocytes. Surprisingly however, astrocytic conditioned medium derived from BAF-treated astrocytes was not able to affect endothelial properties, suggesting that a reciprocal crosstalk between endothelial cells and astrocytes is needed to observe the described effects. Indeed, the mutual interaction among different cellular components of the BBB has been largely suggested and is receiving growing attention.

BAF easily crosses the BBB and, once in the CNS, it interacts with astrocytes expressing S1P1 receptors. In our hands, the reduced PBMCs migration we observed with BAF was mediated by S1P1 as it was mostly prevented by blockade of this receptor subtype with the selective antagonist NIBR. In vitro studies reported that BAF-mediated S1P1 modulation in astrocytes prevented NF-κB nuclear translocation and induced the activation of Nrf2, thus reducing neuronal damage (Colombo et al. 2020). We here confirm that BAF pretreatment reduced T&I-driven inflammation in astrocytes as it blunted enhanced phosphorylation of NF-κB and the consequent expression and activation of the NLRP3 inflammasome (He et al. 2016). Accordingly, mRNA upregulation of the inflammatory cytokine IL-6 and of the chemokines IL-8 and CCL2, all critically regulated by NF-κB, was prevented. All these factors are well known to induce BBB damage. In particular, IL-6 induces sustained endothelial cell loss due to the activation of the JNK-Stat-3 signaling (Alsaffar et al. 2018), whereas the main role of chemokines, or chemoattractive cytokines, is to trigger adhesion and migration of lymphocytes through the endothelial barrier. IL-8 is further involved in angiogenesis and endothelial growth in tumors and wound healing (Li et al. 2003) and CCL2 influences several cellular functions beyond its chemotactic activity. We decided to point our attention to BAF modulation of CCL2, as this chemokine has been already linked to BBB damage in several inflammatory and infectious disorders, such as atherosclerosis and MS (Gschwandtner et al. 2019), and also to S1P signaling (Li et al. 2014). More importantly and in line with our data, CCL2 seems to be mostly overexpressed in perivascular astrocytes and positively correlates with the number of infiltrated T cells in the area, as described in the inflamed parenchyma of human glioma (Carrillo-de Sauvage et al. 2012), in animal models of traumatic brain injury as well as in biopsies of patients affected by MS (Glabinski et al. 1996; Van Der Voorn et al. 1999; Mahad and Ransohoff 2003). Addition of CCL2 prevented BAF-induced effect on PBMCs migration through our in vitro barrier model suggesting that the reduction of CCL2 induced by BAF may be responsible for the observed effect. More specifically, CCL2 can influence leukocytes infiltration inducing a marked endothelial expression of the adhesion molecules ICAM-1 (Kawai et al. 2009). We now report that BAF negatively controls the T&I-induced overexpression of ICAM-1, but this effect is totally overcome when CCL2 levels are rescued by exogenous addition of the chemokine. Of note, CCL2 effect was blocked by CCR2 blockade, confirming that inhibition of the CCL2/CCR2 axis contributes to BAF effect on peripheral cell migration through the barrier. In addition, intracellular redistribution of the adhesion molecule VE-cadherin was impaired in CCL2-treated cells. VE-cadherin, recognized as the main adhesion mechanism for endothelial cell junctional contacts (Vestweber 2008), appears mainly involved in PBMCs transendothelial migration (Shaw et al. 2001). S1P, mostly through S1P1 signaling, is known to prevent VE-Cadherin rearrangement, increasing its stabilization at endothelial cell junctions (Argraves et al. 2010; Shepherd et al. 2017). In our settings, BAF was able to prevent VE-cadherin internalization, but in the presence of the CCL2, BAF lost its ability to counteract T&I-induced VE-cadherin shuttling from the cell membrane to the cytosol to allow leukocyte migration, suggesting, once again, a key role for this astrocyte-derived chemokine. Finally, a correlation already exists between S1P signaling and expression of CCL2. In a mouse model of intra cerebral hemorrhage, S1P3 activation has been shown to increase the expression of CCL2 and inhibition of the S1P3-CCL2-p38MAPK signaling pathway reduced BBB damage and leukocytes adhesion, stabilizing junctional tightness and decreasing ICAM-1 expression (Xu et al. 2021). Although these results may appear in apparent contrast with our findings, it is well established that BAF acts selectively on S1P1 and S1P5, and different S1P receptors are known to mediate different and even contrasting effects (Prager et al. 2015). More specifically, activation of astrocytic S1P3 has been shown to contribute to impaired barrier function through the release of IL-6 and CCL2 (Gril et al. 2018).

In conclusion, our data show that BAF, besides its reported effect on the stability and reduced permeability of the astrocyte/endothelial barrier (Spampinato et al. 2021), is also able to reduce PBMCs transmigration, an effect that involves the reduction of CCL2 released by astrocytes and acting on endothelial cells. Results strengthen the concept of a strict crosstalk between the two main cellular components of the BBB, underlining also the need for a reciprocal interaction and their co-existence in the same environment.

## Data Availability

Data transparency.
